# Melittin Inhibits Colorectal Cancer Growth and Metastasis by Ac-Tivating the Mitochondrial Apoptotic Pathway and Suppressing Epithelial–Mesenchymal Transition and Angiogenesis

**DOI:** 10.3390/ijms252111686

**Published:** 2024-10-30

**Authors:** Kangli Wang, Lingchen Tao, Meifei Zhu, Xinyu Yu, Yuanyuan Lu, Bin Yuan, Fuliang Hu

**Affiliations:** College of Animal Sciences, Zhejiang University, Hangzhou 310058, China; 12017029@zju.edu.cn (K.W.); lingchentao@163.com (L.T.); meifeizhu@zju.edu.cn (M.Z.); yuxinyu@zju.edu.cn (X.Y.); 0914219@zju.edu.cn (Y.L.); yuan_bin322@zju.edu.cn (B.Y.)

**Keywords:** melittin, colorectal cancer, apoptosis, mitochondrial apoptotic pathways, metastasis, EMT, angiogenesis

## Abstract

Melittin has previously been found to have a positive effect on colorectal cancer (CRC) treatment, one of the most difficult-to-treat malignancies, but the mechanism by which this effect occurs remains unclear. We evaluated melittin’s pro-apoptotic and anti-metastatic effects on CRC in vitro and in vivo. The results showed that melittin-induced mitochondrial ROS bursts decreased ΔΨm, inhibited Bcl-2 expression, and increased Bax expression in both cells and tumor tissues. This led to increased mitochondrial membrane permeability and the release of pro-apoptotic factors, particularly the high expression of Cytochrome C, initiating the apoptosis program. Additionally, through wound-healing and transwell assays, melittin inhibited the migration and invasion of CRC cells. In vivo, the anti-metastatic effect of melittin was also verified in a lung metastasis mouse model. Western blotting and immunohistochemistry analysis indicated that melittin suppressed the expression of MMPs and regulated the expression of crucial EMT markers and related transcription factors, thereby inhibiting EMT. Furthermore, the melittin disrupts neovascularization, ultimately inhibiting the metastasis of CRC. In conclusion, melittin exerts anti-CRC effects by promoting apoptosis and inhibiting metastasis, providing a theoretical basis for further research on melittin as a targeted therapeutic agent for CRC.

## 1. Introduction

Colorectal cancer (CRC) is a significant global public health challenge, with high incidence and mortality rates despite advances in treatment. In 2018, CRC accounted for about 10.2% of cancer cases and 9.2% of cancer-related deaths worldwide, according to the International Agency for Research on Cancer [1]. Alarmingly, its incidence is increasingly affecting younger populations, particularly those under 50 [2,3]. Expanding the scope of public screening programs is crucial for reducing CRC-related mortality [4]. Furthermore, traditional treatment methods, such as surgical resection, adjuvant chemotherapy, and radiotherapy, often carry significant risks. These treatments may be associated with poor prognoses, severe side effects, and issues of drug resistance. All of these factors hinder the effectiveness of CRC treatment. Therefore, exploring novel therapeutic strategies represents another critical approach. Beyond conventional chemotherapy, emerging treatments for CRC include immunotherapy [5,6,7], novel nanomedicine approaches [8], therapies targeting CRC stem cells [9], and bacterial therapies [10]. Therefore, the urgent need for more effective therapeutic strategies for CRC warrants further investigation.

Melittin, the primary component of honeybee venom (*Apis mellifera*), is a naturally occurring antitumor peptide with demonstrated efficacy in treating various cancers [11,12,13]. As an amphiphilic, 26-residue linear cationic peptide (GIGAVLKVLTTGLPALISWIKRKRQQ-CONH_2_), melittin’s amphiphilic nature facilitates its penetration of the phospholipid bilayer of mammalian cells, leading to the leakage of intracellular contents [14,15,16]. Additionally, six positive charges enhance melittin’s preferential binding to tumor cell membranes over normal ones [17,18]. Melittin has demonstrated antitumor properties in various cancers through multiple mechanisms, including disrupting cell membranes [16], arresting the cell cycle at the G1 phase and inhibiting proliferation [19], inducing endoplasmic reticulum stress and promoting apoptosis [20], activating autophagy and the unfolded protein response [21], and triggering the mitochondrial apoptotic pathway to inhibit cancer growth [22,23]. Additionally, suppression of migration and invasion represents another critical mechanism by which melittin exerts its antitumor effects [24,25]. These studies have highlighted the remarkable anti-tumor impacts of melittin. Further investigation into how melittin promotes apoptosis and exhibits anti-metastatic properties in CRC could provide new insights for developing melittin-based therapeutic strategies for CRC.

Given that mitochondrial-dependent apoptosis is one of the primary pathways of cell death and that previous studies have not examined whether melittin promotes apoptosis in CRC cells by activating this pathway, we hypothesize that melittin induces apoptosis in CRC cells through the mitochondrial apoptotic pathway. Mitochondrial dysfunction is frequently observed in malignant tumors, and the intrinsic mitochondrial apoptotic pathway is a crucial mechanism of cell apoptosis [26]. Mitochondria, essential intracellular organelles, play a central role in distributing energy metabolites, maintaining and conducting membrane potential, and delivering newly synthesized ATP to various cellular regions [27]. Dysregulated reactive oxygen species (ROS) metabolism and alterations in ΔΨm occur at the early stages of apoptosis in cancer cells [28,29]. Upon the activation of MPT, Cytochrome C is released from mitochondria into the cytoplasm, triggering a caspase cascade that induces DNA damage and cellular structural breakdown [30]. Previous studies have indicated that stimulating ROS production, disrupting ΔΨm, and upregulating the expression of Cytochrome C, Bax, caspase-3, and caspase-9 are critical features in inducing mitochondrial-dependent apoptosis in lung cancer cells [31,32]. Furthermore, melittin induces apoptosis in lung cancer cells through the mitochondrial pathway, characterized by triggering mitochondrial ROS bursts, inducing ΔΨm depolarization, and activating the expression of proteins associated with the mitochondrial apoptosis pathway [33].

The epithelial–mesenchymal transition (EMT) activation and angiogenesis processes are primarily involved in CRC metastasis. Therefore, it is essential to investigate the anti-metastatic mechanisms of melittin on CRC from the perspectives of EMT and angiogenesis. In EMT, epithelial cells lose their characteristics and transition into mesenchymal cells, resulting in cell adhesion molecules and cytoskeleton alterations, promoting cell motility and invasiveness [34]. Tumor cells acquire metastatic properties by activating EMT, which enables them to invade surrounding tissues and colonize distant organs through extensive angiogenesis [35,36]. Activation of cadherin serves as a notable indicator of EMT initiation, with the downregulation or degradation of E-cadherin and upregulation of N-cadherin disrupting intercellular junctions in metastatic cancer stem cells, thereby facilitating cell migration and invasion [37,38]. The Snail and Twist transcription factors frequently regulate E-cadherin, an epithelial marker, and N-cadherin, a mesenchymal marker, which are critical in EMT activation during cancer progression [34]. Snail upregulates the expression of E-cadherin and claudins while downregulating vimentin and fibronectin [39,40]. Similarly, Twist suppresses E-cadherin expression while promoting N-cadherin expression [41,42]. Moreover, during EMT initiation, Snail upregulates matrix metalloproteinases (MMPs) such as MMP2 and MMP9, which catalyze the degradation of extracellular matrix (ECM) substrates, enhancing the invasion of blood vessels and lymphatics [43]. Therefore, the impact of melittin on EMT and angiogenesis is a crucial area of research in terms of its potential anti-CRC effects.

Previous studies have shown that the inhibitory effect of melittin on CRC and its mechanism are not comprehensive. Therefore, we speculate that inhibiting colorectal cancer progression by melittin involves mitochondrial-dependent apoptosis, epithelial–mesenchymal transition (EMT), and angiogenesis. In our study, we first investigated the inhibition capacity of melittin in CRC development through induced apoptosis and suppressed lung metastasis. Further, melittin induced tumor apoptosis and inhibited tumor growth through the endogenous mitochondrial pathway. Meanwhile, Melittin significantly inhibited the invasion and metastasis of CRC by regulating the EMT process and suppressing angiogenesis. Our study advances the exploration of the inhibitory effects of melittin on CRC and its mechanisms, establishing a foundation for investigating novel therapeutic strategies associated with melittin.

## 2. Results

### 2.1. Melittin Suppresses CRC Cells’ Viability in a Concentration- and Time-Dependent Manner

**Figure 1:**
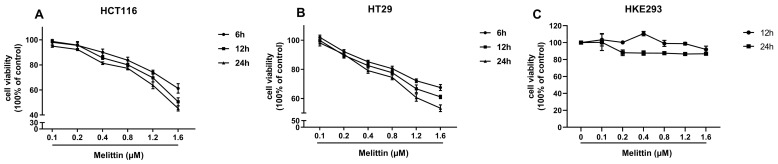
Melittin suppresses the viability of CRC cells. CRC cells were seeded in 96-well plates and treated with melittin for 6, 12, and 24 h. Each concentration of melittin was tested in 8 replicates. (**A**,**B**) the effects of melittin on the viability of HCT116 and HT29 cells, respectively, as measured by the CCK-8 assay; (**C**) the effect of melittin on the viability of HEK293 cells.

To avoid significant damage to normal cells from high concentrations of melittin, we assessed the effects of different concentrations of melittin on the cell viability of CRC cell lines (HCT116, HT29) and Human Embryonic Kidney 293 cells (HEK293). The results showed that melittin reduced the viability of CRC cells in a concentration- and time-dependent manner, while it had no significant effect on HEK293 cells at 12 and 24 h (Figure 1A–C). This suggests that melittin may induce tumor cell death through pathways other than disrupting the phospholipid bilayer. Notably, HCT116 cells treated with 0.1 μM and 1.6 μM melittin for 24 h exhibited cell viabilities of 95.04% and 45.26%, respectively, whereas HT29 cells showed 98.17% and 53.35%. This demonstrates an apparent decrease in cell viability with increasing melittin concentrations. Moreover, when treated with the same concentration of melittin (1.6 μM) for 6 and 24 h, tumor cell viability decreased in a time-dependent manner. Specifically, HCT116 cells exhibited viabilities of 61.27% and 45.26% after 6 and 24 h, respectively, while HT29 cells showed 67.55% and 53.35% at the same time points. These findings indicate that melittin suppresses CRC cell viability in both a concentration- and time-dependent manner.

### 2.2. Melittin Induces Apoptosis in CRC Cells Both In Vivo and In Vitro

**Figure 2:**
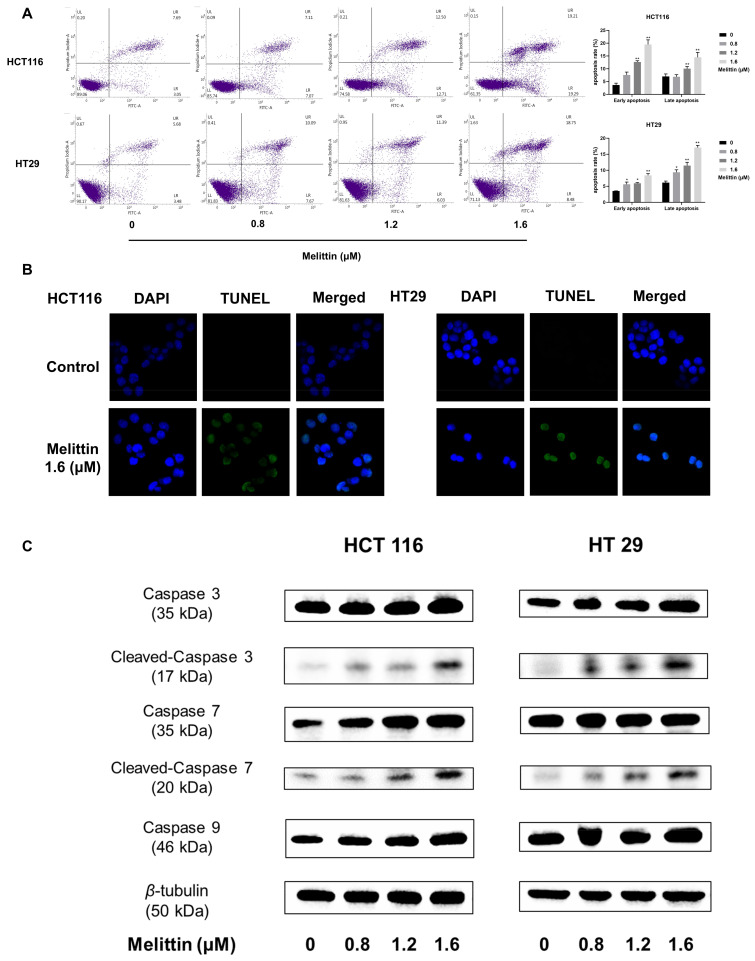
Melittin induces apoptosis in CRC cells by activating the caspase cascade. (**A**) Annexin V-FITC/PI staining was used to assess the extent of apoptosis induced by melittin in CRC cells; (**B**) TUNEL staining images were captured after treating CRC cells with melittin for 24 h; (**C**) the expression levels of Caspase 3, Cleaved-caspase 3, Caspase 7, Cleaved-caspase 7 and Caspase 9 in CRC cells were analyzed by Western blot. Quantification of the bands was performed, and statistical analysis was conducted to determine significant differences (Supplementary Figure S1). Statistical significance: *, *p* < 0.05, **, *p* < 0.01.

We examined the effect of melittin on apoptosis in CRC cells in vitro using Annexin V-FITC/PI staining, Terminal deoxynucleotidyl transferase-mediated dUTP nick-end labeling (TUNEL) staining, and Western blot, respectively. As depicted in Figure 2A, melittin treatment at concentrations of 1.2 μM and 1.6 μM significantly enhanced both early and late apoptosis in HCT116 and HT29 cells, with apoptotic cell proportions reaching 33.94% and 25.52%, respectively, at 1.6 μM melittin. TUNEL staining revealed the onset of nuclear shrinkage and DNA fragmentation in both cell lines at this concentration (Figure 2B). Western blot analysis further demonstrated increased expression of apoptosis-related proteins in melittin-treated HCT116 and HT29 cells, including Caspase 3, Cleaved-Caspase 3, Caspase 7, Cleaved-Caspase 7, and Caspase 9 (Figure 2C). In particular, caspase 9 was increased by 1.49-fold and 1.28-fold (*p* < 0.05), respectively (Supplementary Figure S1). Therein, Cleaved-Caspase 3 is an active form related to apoptosis execution, activating the poly ADP-ribose polymerase (PARP), inducing the formation of apoptotic bodies, and chromosomal DNA fragmentation [44,45]. Moreover, as a crucial initiator of caspase involved in the intrinsic mitochondrial apoptosis signaling pathway [46], the increased expression level of caspase 9 raised our concern.

**Figure 3:**
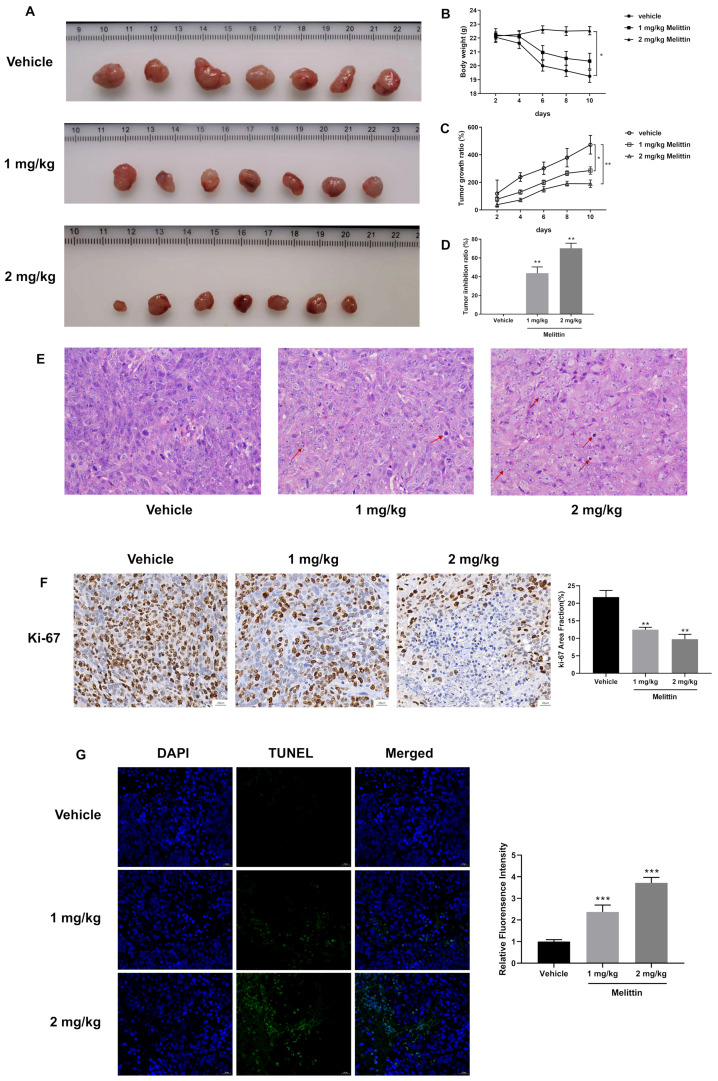
Melittin suppresses tumor growth in a subcutaneous heterograft tumor model using BALB/c nude mice. Mice were inoculated with 1 × 10^7^ HCT116 cells; when tumor volumes reached 80 mm^3^, intratumoral melittin injections were administered at 1 mg/kg or 2 mg/kg every two days. (**A**) Tumors were collected from nude mice (*n* = 8) after a total of five treatments; (**B**) body weight changes were monitored over 10 days, with noticeable weight loss observed as the tumors progressed; (**C**) tumor volume changes were measured and statistically analyzed over 10 days; (**D**) the tumor inhibition ratio was calculated based on final tumor weight measurements; (**E**) H&E stained sections were utilized to assess the histopathological features of the tumor tissues. The area indicated by the arrow shows inflammatory cell infiltration, primarily consisting of lymphocytes and plasma cells; (**F**) immunohistochemical (IHC) analysis for Ki-67 indicated a significant reduction in tumor proliferation in melittin-treated mice; (**G**) TUNEL fluorescence intensity was markedly increased in the tumors of melittin-treated mice compared to the control group, further confirmed by statistical analysis, demonstrating melittin’s pro-apoptotic effects in tumor cells (*n* = 4). Statistical significance: *, *p* < 0.05, **, *p* < 0.01, ***, *p* < 0.001.

In vivo, melittin exhibited significant anti-tumor activity in a subcutaneous xenograft mouse model, as illustrated in Figure 3A and Supplementary Figure S2. Tumor growth and inhibition ratios (Figure 3C,D) demonstrated melittin’s efficacy, with a marked reduction in tumor-associated weight loss in high-dose melittin-treated mice (*p* < 0.05) (Figure 3B). Histopathological examination of H&E-stained tumor sections revealed notable differences between melittin-treated and control tumors (Figure 3E). Tumors from control mice displayed tight, well-structured tissue with large central nuclei and minimal inflammatory cell infiltration. In contrast, melittin-treated tumors showed a loose structure, increased intercellular spaces, cell shrinkage, nucleolus condensation, and abundant apoptotic bodies, with pronounced inflammatory cell infiltration, predominantly macrophages, and plasma cells. These effects were more evident in the high-dose melittin group. Additionally, a significant reduction in Ki-67 protein expression (*p* < 0.01) (Figure 3F) indicated a decrease in tumor cell proliferation in response to melittin treatment. The TUNEL fluorescence results (Figure 3G) further corroborated these findings, showing extensive apoptotic cell areas in melittin-treated tumors, with a higher quantity of apoptotic cells in the high-dose group (*p* < 0.001).

### 2.3. Melittin Induces Apoptosis in CRC Through Mitochondrial Pathway

**Figure 4:**
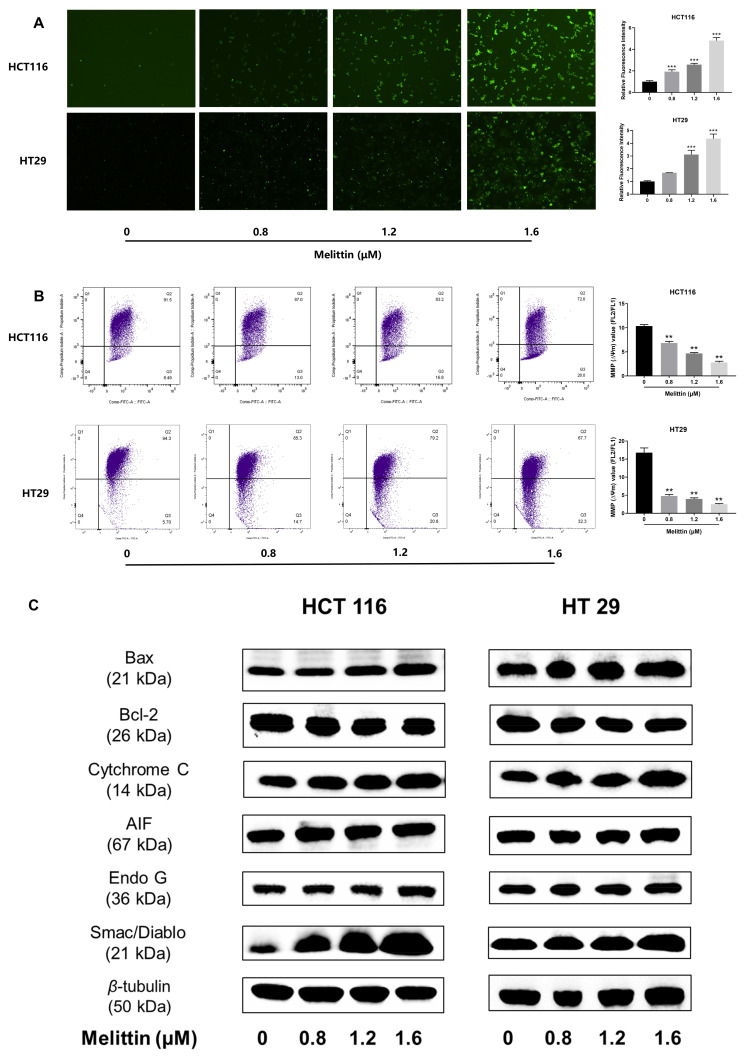
Melittin induces mitochondrial pathway-mediated apoptosis in vitro. HCT116 and HT29 cells were treated with various melittin concentrations for 24 h, and the effects were evaluated using ROS measurement, JC-1 staining, and Western blot analysis. (**A**) Melittin treatment led to an increase in ROS levels in CRC cells; (**B**) melittin-induced changes in ΔΨm, as demonstrated by JC-1 staining; (**C**) protein expression levels of Bax, Bcl-2, Cytochrome C, AIF, Endo G, and Smac/Diablo were analyzed by Western blot, and the data were quantified and statistically analyzed (Supplementary Figure S3). Statistical significance: **, *p* < 0.01, ***, *p* < 0.001.

To investigate whether melittin induces apoptosis in CRC cells through mitochondrial mechanisms, we first conducted a mitochondrial ROS assay on HCT116 and HT29 cell lines treated with melittin. The images and statistical analysis (Figure 4A) indicated that melittin treatment significantly increased intracellular ROS levels, with ROS levels being markedly higher at concentrations of 1.2 μM and 1.6 μM compared to untreated cells (*p* < 0.001). The relative fluorescence intensity peaked at 4.82 ± 0.28 in HCT116 cells and 4.36 ± 0.37 in HT29 cells (*p* < 0.001, *p* < 0.001). Elevated ROS levels in tumor cells led to alterations in ΔΨm, influencing mitochondrial permeability transition (MPT). JC-1 staining was used to assess ΔΨm, and the results showed that the ΔΨm in melittin-treated HCT116 cells decreased by 72.94% compared to the control group, while the ΔΨm in HT29 cells decreased by 85.03% (Figure 4B) (*p* < 0.01). Further analysis of Bcl-2 family proteins (Bcl-2, Bax) showed that melittin treatment induced MPT (Figure 4C), indicating compromised mitochondrial membrane integrity and activation of the apoptotic pathway, as evidenced by the reduced ΔΨm. Consequently, various mitochondrial proteins, including Cytochrome C, were released into the cytoplasm.

**Figure 5:**
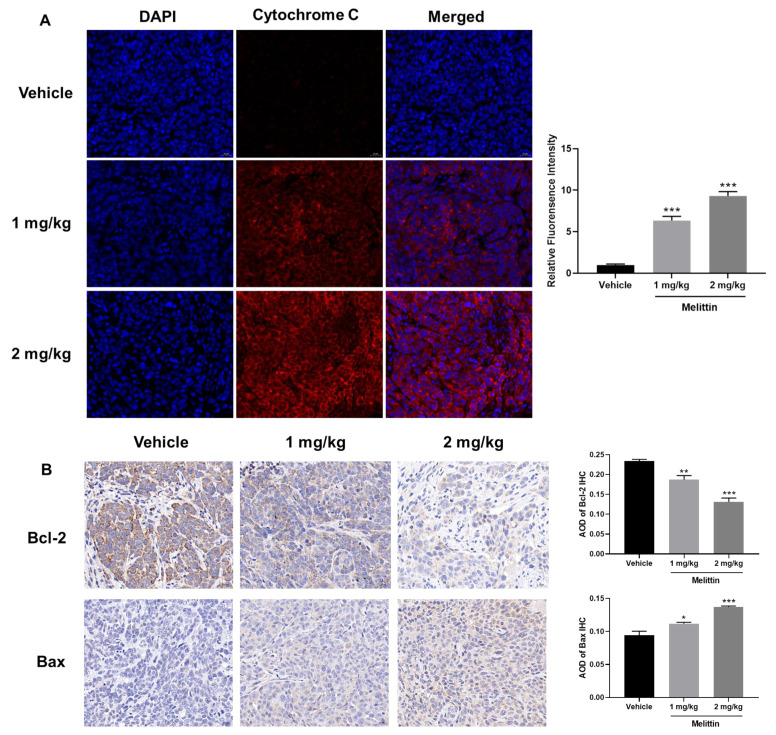
Melittin induces mitochondrial pathway-mediated apoptosis in vivo. (**A**) Melittin treatment led to a significant release of Cytochrome C in tumor tissues from BALB/c mice, visualized as red fluorescence; (**B**) IHC analysis of Bcl-2 and Bax expression levels demonstrated that melittin attenuates tumor progression. All data were quantified and statistically analyzed to assess significant differences. Statistical significance: *, *p* < 0.05, **, *p* < 0.01, ***, *p* < 0.001.

Subsequently, we measured the expression levels of additional proteins involved in the mitochondria-mediated apoptotic pathway, including Bcl-2 family proteins (pro-apoptotic protein, Bax; anti-apoptotic protein, Bcl-2), as well as Cytochrome C, AIF, Endo G, and Smac/Diablo. In melittin-treated tumor cells, we observed a significant increase in the expression levels of Bax, Cytochrome C, AIF, Endo G, and Smac/Diablo, accompanied by a notable decrease in Bcl-2 expression (Figure 4C). These results indicate that melittin induces apoptosis by activating the intrinsic mitochondrial pathway, which is further corroborated by our in vivo analyses. Immunofluorescent staining of tumor tissues from mice demonstrated that compared to the control group, the level of cytochrome C was significantly increased in the melittin treatment group (*p* < 0.001), with the fluorescence intensity in the high-dose group being 9.3-fold that of the control group (Figure 5A), suggesting enhanced expression and release of Cytochrome C from mitochondria in response to melittin treatment. Additionally, histological analysis of tumor tissues revealed that melittin treatment resulted in a significant reduction in Bcl-2 levels and a concomitant increase in Bax levels (*p* < 0.05, *p* < 0.01, *p* < 0.001) (Figure 5B).

### 2.4. Melittin Inhibits Invasion and Metastasis of CRC In Vivo and In Vitro

**Figure 6:**
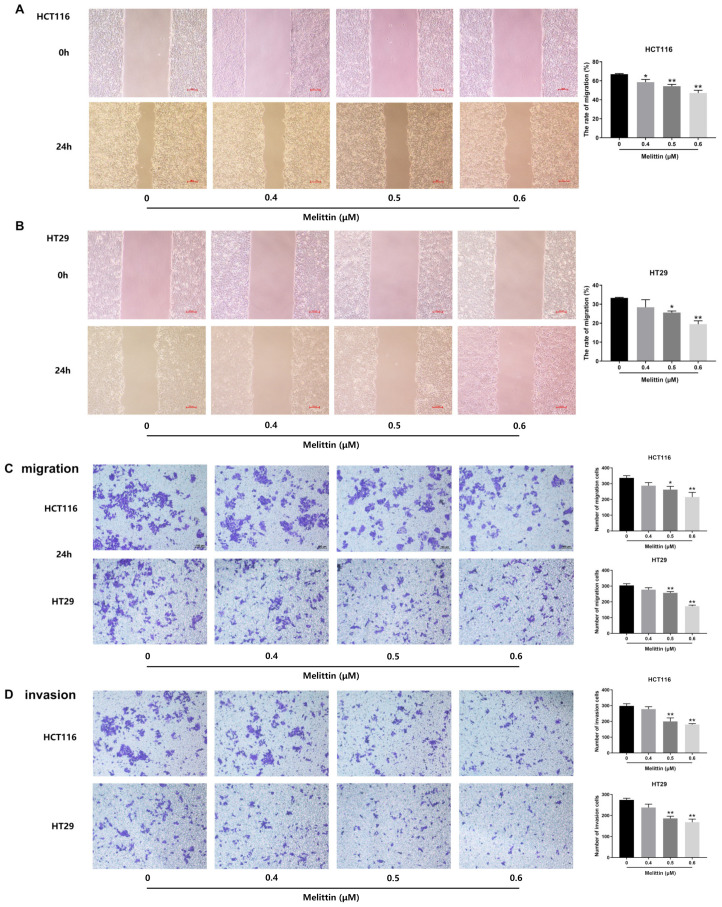
Melittin inhibits migration and invasion of CRC cells. HCT116 and HT29 cells were treated with different melittin concentrations for 24 h (*n* = 4). (**A**,**B**) wound-healing assays revealed that melittin significantly slowed the lateral migration rate of CRC cells; (**C**) in transwell migration assays, melittin markedly reduced the longitudinal mobility of CRC cells over a 24 h period; (**D**) in the transwell invasion assay, melittin inhibited cell invasion by significantly blocking the movement of CRC cells through Matrigel, over 24 h. All data were quantified and statistically analyzed to assess significant differences. Statistical significance: *, *p* < 0.05, **, *p* < 0.01. Scale bar: 100 μm.

To mitigate the impact of apoptosis induced by high melittin concentrations on tumor cells, we employed lower melittin concentrations for migration and invasion assays. Lateral migration of tumor cells treated with melittin was assessed using a wound-healing assay. As shown in Figure 6A,B, the lateral migration velocity of melittin-treated tumor cells was significantly reduced compared to untreated cells (*p* < 0.05, *p* < 0.01). Specifically, HCT116 and HT29 cells treated with 0.6 μM melittin for 24 h exhibited a decrease in migration rate by 47.16% and 19.49%, respectively. Longitudinal migration capacity was evaluated, as illustrated in Figure 6C, where melittin at concentrations of 0.5 μM or 0.6 μM significantly inhibited tumor cell migration, as determined by counting the cells traversing transwell inserts’ polycarbonate membrane (*p* < 0.05, *p* < 0.01). Additionally, the invasive potential of tumor cells, measured by their ability to cross the matrigel-coated polycarbonate membrane, showed a significant reduction in the number of invading cells following treatment with 0.5 μM or 0.6 μM melittin compared to the control group (*p* < 0.01). In particular, treatment with 0.6 µM melittin decreased invasive cells by 39.6% for HCT116 cells and 38.76% for HT29 cells (Figure 6D). These findings indicate that melittin suppresses tumor cell migration and invasion in vitro.

**Figure 7:**
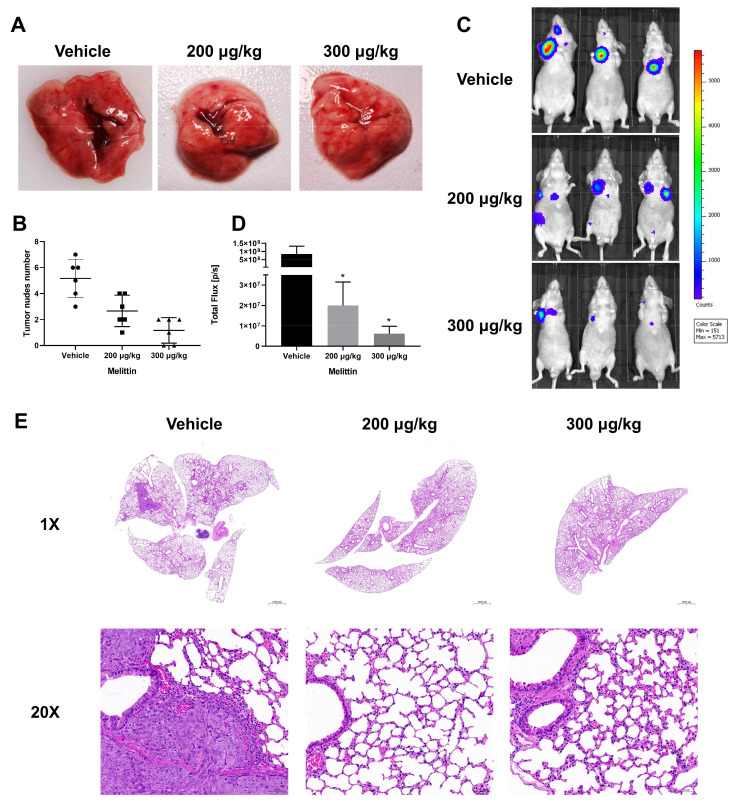
Melittin inhibits the formation of pulmonary metastatic nodules in nude mice with CRC. Approximately 1 × 10^7^ HCT116 ^luc^ cells were injected into BALB/c mice via the tail vein to establish a lung metastasis model. Mice were subsequently treated with melittin or PBS solution seven times. (**A**,**B**) Pulmonary nodules were visibly more numerous in the control group than in the melittin-treated group. The number of pulmonary nodules in each group was quantified. In panel (**A**), the area indicated by the arrow is the metastatic lesion in the lung; (**C**,**D**) in vivo imaging was performed using the IVIS imaging system, with fluorescence density quantified and statistically analyzed; (**E**) pathological analysis of lung tissue was conducted using H&E-stained sections observed at 1× and 20× magnification. Bar: 1000 µm (1×), 50 µm (20×). The control group’s lungs exhibited a denser tumor structure compared to the melittin-treated group. Statistical significance: *, *p* < 0.05.

To further evaluate melittin’s efficacy in inhibiting CRC cell metastasis to the lung in vivo, nude mice were treated with two doses of melittin in a lung metastasis model. Notable pulmonary metastatic lesions were observed in the control group, whereas such lesions were nearly absent in melittin-treated mice (Figure 7A). Furthermore, a statistical analysis of pulmonary metastatic nodules demonstrated a reduction in their number due to melittin treatment (Figure 7B). In vivo imaging of luciferase activity showed that the signal in melittin-treated mice decreased by over 97% compared to the control group. (*p* < 0.05) (Figure 7C,D). Upon observing the H&E-stained pathological sections of lung tissue under 1× magnification, we noticed the dense structure of the pulmonary metastatic lesions and higher infiltration in the control mice (Figure 7E). A more detailed examination at 20× magnification revealed that the tumor borders in the control group were characterized by extensive neovascularization, suggesting increased tumor invasiveness. Neovascularization is essential for tumor growth and dissemination, providing necessary oxygen and nutrients and creating pathways for metastasis [47]. In contrast, the lung tissue from melittin-treated mice displayed clear alveolar and airway structures with minimal tumor foci, suggesting a lower risk of metastasis (Figure 7E).

### 2.5. Melittin Suppresses Invasion and Metastasis by Inhibiting EMT Process

**Figure 8:**
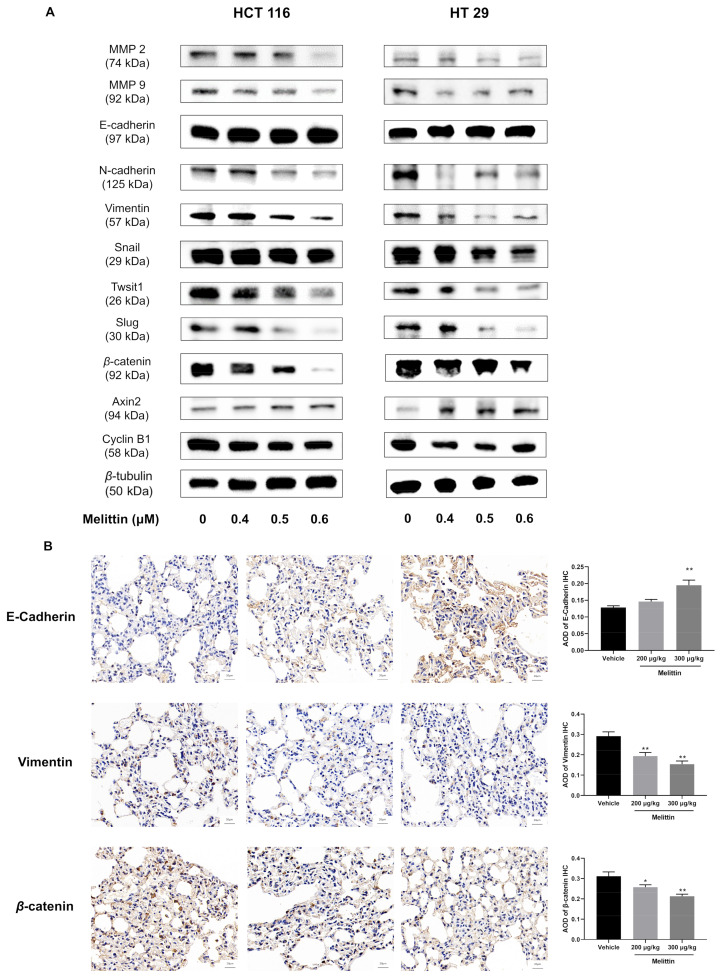
Melittin reverses EMT by inhibiting the *β*-catenin signaling pathway in vivo and in vitro. (**A**) Western blot analysis revealed changes in the protein levels of MMP, EMT-related proteins in CRC cells treated with melittin for 24 h (Supplementary Figure S4); (**B**) IHC analysis quantitatively assessed the expression levels of E-cadherin, Vimentin, and *β*-catenin. All data were quantified and statistically analyzed to determine significant differences. Statistical significance: *, *p* < 0.05, **, *p* < 0.01.

Abnormal activation of the EMT process is a crucial step in tumor development and metastasis. EMT reduces cell-to-cell adhesion and packing density, thereby facilitating metastasis. To evaluate whether melittin inhibits tumor cell invasion and metastasis by interfering with EMT progression, we examined the expression of EMT-associated proteins, including MMPs (MMP2, MMP9), E-cadherin, N-cadherin, Vimentin, and related regulatory protein, in both in vivo and in vitro models. Western blot analysis revealed that melittin significantly reduced the expression levels of MMP2 and MMP9 in a dose-dependent manner, indicating impaired tumor cell movement, consistent with the results from wound-healing and transwell assays (Figure 8A). Additionally, E-cadherin expression increased in melittin-treated tumor cells, while N-cadherin and Vimentin levels decreased (Figure 8A). Notably, high-dose melittin treatment significantly upregulated E-cadherin, with an increase of 51.75% compared to the control group (*p* < 0.01) (Figure 8B). Conversely, Snail, Slug, and Twist1, as upstream regulators of EMT markers, were significantly reduced in melittin-treated tumor cells compared to untreated cells (Figure 8A). These changes suggest that melittin inhibits tumor cell migration and invasion by reversing EMT.

In addition, compared to the control group tumor cells, melittin treatment resulted in a reduction in *β*-catenin protein levels and an increase in Axin2 protein levels, reminding us that melittin may regulate the EMT process through the *β*-catenin signaling pathway (Figure 8A). Correspondingly, IHC analysis of tumor tissue from mice confirmed a significant decrease in *β*-catenin protein expression following melittin treatment (*p* < 0.01) (Figure 8B). Additionally, the reduced level of Cyclin B1, a downstream regulatory protein of the *β*-catenin signaling pathway in melittin-treated tumor cells, further supports the involvement of this pathway in modulating the EMT process (Figure 8A).

### 2.6. Melittin Attenuates Angiogenesis In Vitro

**Figure 9:**
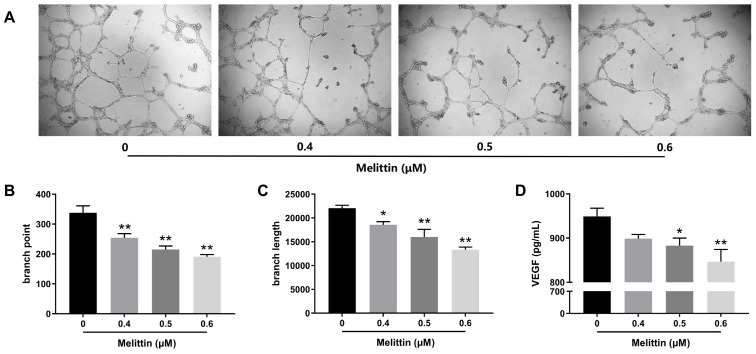
Melittin inhibits angiogenesis in vitro. (**A**) The effect of melittin on the tube formation ability of HUVECs was assessed. HUVECs were cultured on an ECM and treated with melittin for 16 h; (**B**,**C**) the number of branch points and branch length of tubes formed were quantified in the tube formation assay; (**D**) the concentration of VEGF in HUVECs varies with the dosage of melittin. Statistical significance: *, *p* < 0.05, **, *p* < 0.01.

Angiogenesis is critical for tumor growth and metastasis, making it a key focus in evaluating melittin’s inhibitory effects on CRC. As illustrated in Figure 9A, melittin disrupted tube formation in human umbilical vein endothelial cells (HUVECs) at varying concentrations. Statistical analyses of branch points and branch lengths revealed that melittin significantly reduced the number of branch points and shortened branch lengths at all tested concentrations (*p* < 0.05, *p* < 0.01) (Figure 9A,B). Specifically, treatment with 0.6 μM melittin resulted in a 43.4% decrease in branch points and a 39.56% reduction in branch length compared to the control group (*p* < 0.01). Moreover, as shown in Figure 9C, Vascular endothelial growth factor (VEGF) secretion levels in HUVECs treated with higher melittin concentrations (0.5 μM and 0.6 μM) were significantly lower than in the control group (*p* < 0.05, *p* < 0.01), indicating specific reductions of 6.95% and 10.76%, respectively.

## 3. Discussion

Numerous studies confirm that bee venom inhibits various cancers, including pancreatic, prostate, lung, glioblastoma, breast, and CRC [48,49,50,51,52,53]. Melittin, the primary component of bee venom, is mainly responsible for its anti-tumor effects. It has been shown to induce apoptosis in breast- and lung- cancer cells by downregulating the TGF-*β*/ERK signaling pathway and reversing the EMT process in breast cancer by inhibiting the HIF-1*α* pathway [54,55,56]. In liver cancer, melittin reduces CoCl₂-induced migration, invasion, and hypoxia-induced vasculogenic mimicry by decreasing HIF-1*α* and reversing EMT markers [57]. Given CRC’s high incidence and postoperative metastasis rates, melittin’s inhibitory effects on CRC are essential to explore. Our data showed that melittin suppressed CRC cell proliferation in a time- and dose-dependent manner without affecting normal cells at a low concentration (Figure 1A–C), aligning with previous studies [58]. Reports indicate melittin activates the unfolded protein response and autophagy, elevating UPR markers CHOP, XBP-1s, and LC3-*β*II while reducing P62, critical mechanisms in its anti-CRC action [21]. Additionally, melittin induces ER stress-mediated apoptosis by disrupting calcium homeostasis in SW480 cells and mice [20]. Since the mitochondrial apoptosis pathway and EMT are critical for cancer progression, we further explored melittin’s ability to trigger mitochondria-dependent apoptosis and reverse EMT to inhibit CRC growth and metastasis.

The amphipathic nature of melittin leads to non-specific pore formation in cell membranes. However, melittin induces tumor cell death at lower concentrations without affecting normal cells, suggesting that melittin triggers cell death through additional mechanisms. In our study, Annexin V-FITC/PI results showed that higher melittin concentrations correlated with increased tumor cell apoptosis, while TUNEL staining images displayed fluorescence from DNA fragments generated during apoptosis (Figure 2A,B). As key “executor” of apoptosis [59], the expression levels of caspase-3 and caspase-7 in colorectal tumor cells significantly increased after melittin treatment (Figure 2C). In non-small-cell lung cancer cells, melittin has been shown to induce apoptosis by increasing caspase-3 protein expression and Apaf-1 mRNA levels [55]. Additionally, the increased expression of caspase-9 prompted us to investigate whether it induces mitochondrial alterations (Figure 2C), as caspase-9 is the only initiator of caspase implicated in mitochondrion-dependent apoptosis [60].

Our study’s decrease in ΔΨm and excessive ROS production indicate that melittin induces mitochondrial dysfunction in CRC cells, triggering mitochondrial apoptosis (Figure 3A,B), consistent with Li’s report [33]. For rapid proliferation, ROS levels are typically maintained at high levels in tumor cells; however, excessive ROS can cause oxidative stress, cellular damage, and apoptosis [61]. Additionally, ROS bursts are often accompanied by a drop in ΔΨm, which increases mitochondrial membrane permeability, releasing Cytochrome C and other pro-apoptotic factors and activating the mitochondrial apoptosis pathway [62,63]. The changes in Bcl-2 and Bax, regulators of mitochondrial membrane permeability, in melittin-treated CRC cells further support the increased mitochondrial membrane permeability (Figure 4C). Subsequently, this leads to the release of a large number of pro-apoptotic factors such as Cytochrome C, AIF, Endo G, and Smac/Diablo from the mitochondria into the cytosol, as indicated by our Western blot analysis of melittin-treated CRC cells (Figure 4C). In the cytosol, Cytochrome C forms an apoptosome with Apaf-1 and caspase-9, triggering caspase-9 activation and initiating the caspase cascade apoptosis [64], as observed in our study. Additionally, Smac/Diablo suppresses the inhibitor of apoptosis proteins (IAPs), further promoting caspase activation. Meanwhile, AIF and Endo G translocate to the nucleus, executing caspase-independent apoptosis [65]. Our findings suggest that melittin induces mitochondrion-dependent apoptosis by increasing ROS production, reducing mitochondrial membrane permeability, and releasing pro-apoptotic factors, providing a pathway through which melittin suppresses CRC growth.

Impeding tumor cell metastasis is another essential strategy for CRC treatment. In the study of anti-metastatic hepatocellular carcinoma, melittin reduced cell motility and migration, as shown by cell-migration and -invasion assays [13]. Consistent with its effects on prostate cancer [66], our wound-healing and transwell migration assays revealed that melittin significantly inhibited CRC cell migration (Figure 6A–D). Furthermore, an in vitro lung metastasis assay in nude mice confirmed that melittin reduced the number of pulmonary metastatic nodules in CRC (Figure 7B). The reduction of MMP2 and MMP9 protein expression implied decreased degradation of the ECM and basement membrane (Figure 8A), which further impeded cell migration and invasion. This finding is consistent with reports that melittin reduces MMP2 activity and protein levels in glioblastoma cells [51] and inhibits MMP9 expression in breast cancer MCF-7 cells [67].

The epithelial–mesenchymal transition has been postulated as an absolute requirement for tumor migration, invasion, and metastasis [68]. Prognostic observation of CRC patients showed that loss of E-cadherin or overexpression of N-cadherin were correlated with poor prognosis and postoperative metastasis [69,70]. Therefore, we hypothesized that melittin inhibits CRC metastasis by reversing EMT, characterized by persistent plasticity. E-cadherin, an epithelial cell-junction protein for EMT initiation, was significantly increased in melittin-treated tumor cells or mice compared to the control group (Figure 8A,B). The protein expression levels of N-cadherin and Vimentin were repressed in melittin-applied cells, which demonstrates that the epithelial-to-mesenchymal transition is reversed (Figure 8A,B). Moreover, the inhibitory effect of melittin on EMT was confirmed in the anti-metastasis of gastric cancer and liver cancer cells [57,71]. Snail, Slug, and Twist1, key transcription factors that drive EMT, are activated early during EMT in CRC. Their overexpression is strongly associated with increased invasiveness, metastasis, and poor prognosis in CRC [72]. Our results demonstrated a reversal of Snail, Slug, and Twist1 overexpression in melittin-treated CRC cells (Figure 8A), suggesting that melittin inactivates these transcription factors, inhibits EMT, and prevents further tumor cell dissemination from the primary site. Among the various regulators of EMT, the Wnt/*β*-catenin axis has emerged as a versatile modulator [73]. In CRC cells, Wnt promotes Snail expression, while inhibition of Wnt/*β*-catenin signaling reduces Snail expression and impedes EMT [74]. As the core molecule of the Wnt/*β*-catenin signaling pathway, *β*-catenin expression has a remarkable decrease in CRC cells with melittin treatment compared to that in CRC cells free of melittin (Figure 8A,B). During EMT, stabilized *β*-catenin translocates to the nucleus, where it interacts with TCF/LEF transcription factors to upregulate Axin2, a negative feedback regulator of *β*-catenin signaling [75]. The enhancement of Axin2 expression level in melittin-applied CRC cells compared to that in control-group CRC cells suggests that melittin suppresses EMT by inhibiting *β*-catenin signaling (Figure 8A). Furthermore, Cyclin B1, a downstream regulator of the *β*-catenin signaling pathway, was downregulated in melittin-treated CRC cells, potentially contributing to reduced cell proliferation and invasion (Figure 8A). For tumor cells to successfully metastasize to distant sites after detaching from the primary location, the support of newly formed blood vessels is indispensable.

Tumor angiogenesis is another pivotal factor in tumor metastasis and growth, commonly called the “angiogenic switch,” providing oxygen and nutrients to tumor cells and facilitating metastatic cell entry into the bloodstream [76]. VEGF, one of the most critical cytokines regulating angiogenesis, directly acts on endothelial cells to induce angiogenesis, particularly in CRC [36]. In tube formation assays, we observed a collapse in the tube formation of HUVECs induced by melittin treatment, which was accompanied by a decrease in VEGF secretion in the culture media (Figure 9A–D), suggesting that melittin exerts an inhibitory effect on angiogenesis. This effect was further corroborated by the pathological examination of lung tissue from a nude mouse model, where reduced neovascularization was observed in melittin-treated lung tumors (Figure 7E). Previous reports have also demonstrated melittin’s ability to inhibit tumor angiogenesis in osteosarcoma by modulating the SDF-1*α*/CXCR4 signaling pathway [77]. Interestingly, overexpression of VEGF has been shown to promote EMT-inducing nuclear translocation of *β*-catenin and upregulating Snail and Twist expression, suggesting a link between angiogenesis and EMT promotion [78]. In our present study, melittin suppressed VEGF secretion and EMT in CRC cells, highlighting its multifaceted role in inhibiting tumor growth and metastasis.

**Figure 10:**
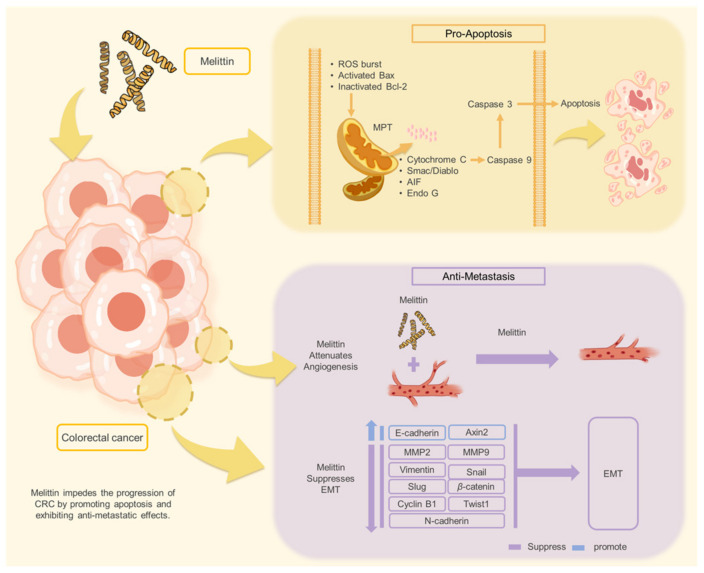
Melittin impedes the progression of CRC by promoting apoptosis and exhibiting anti-metastatic effects.

In conclusion, our study demonstrates that melittin inhibits CRC growth and metastasis through two primary mechanisms: induction of apoptosis via the mitochondrial apoptotic pathway and suppression of EMT and angiogenesis. Specifically, we found that melittin induces apoptosis in CRC cells by promoting ROS production, increasing mitochondrial membrane permeability, releasing mitochondrial apoptosis-related factors, and activating caspase-dependent and caspase-independent apoptotic pathways in vitro and in vivo. Additionally, our results showed that melittin, at lower concentrations, suppresses CRC cell migration, invasion, and metastasis by inhibiting angiogenesis, modulating the *β*-catenin signaling pathway, regulating EMT-related transcription factors and protein markers, and reversing the EMT process (Figure 10).

These studies indicate that future research should explore new drug formulations and therapeutic approaches targeting CRC based on melittin, emphasizing the precise molecular pathways in enhancing treatment efficacy. A limitation of our study is that we did not address the hemolytic activity associated with melittin administration, which restricts the drug’s therapeutic potential. Addressing the hemolytic issues of melittin and optimizing its effects may provide valuable insights for solving future clinical challenges related to CRC.

## 4. Materials and Methods

### 4.1. Reagents

Melittin was purchased from Aladdin Reagent Co., LTD (Shanghai, China).

### 4.2. Cell Culture and Treatments

CRC cell lines (HCT116, HT29) were purchased from the Chinese Academy of Sciences Cell Bank (Shanghai, China). HEK293 cells were generously provided by Zhejiang Chinese Medical University (Hangzhou, China). The CRC cells were cultured in McCoy’s 5A supplemented with 10% fetal bovine serum (FBS) (Gibco, Waltham, MA, USA) and 1% penicillin-streptomycin. HEK293 cells were cultured in DMEM high-glucose medium supplemented with 10% FBS and 1% penicillin-streptomycin. All cells were maintained in a humidified atmosphere containing 5% CO_2_ at 37 °C. After seeding into plates and incubating for 24 h, the cells were treated with various concentrations of melittin.

### 4.3. Cell Viability Assays

Cell viability was assessed using the Cell Counting Kit-8 (Beyotime Co., Ltd., Nanjing, China) according to the manufacturer’s instructions. A total of 1 × 10^4^ cells were seeded onto 96-well plates (100 μL/well) and then incubated for 24 h. Subsequently, the cells were treated with various concentrations of melittin for 6, 12, and 24 h. After treatment, 10 μL of CCK-8 solution was added to each well, and the cells were incubated for one h. Absorbance was measured at 450 nm using a microplate reader (Bio-Rad, Model 550, Hercules, CA, USA).

### 4.4. Annexin V-FITC/PI Assays

Cell apoptosis was assessed using the Annexin V-FITC/PI apoptosis detection kit (Dojindo Co., Ltd., Kumamoto, Japan). CRC cells were treated with melittin for 24 h, followed by washing with phosphate-buffered saline (PBS), digesting with EDTA-free trypsin, and harvesting by centrifugation. Cells were then resuspended in a binding buffer and incubated with Annexin V-FITC and PI, according to the manufacturer’s protocol. Apoptosis was analyzed using CellQuest 5.1 software (BD Biosciences, Franklin Lakes, New Jersey, NJ, USA) and flow cytometry (FACSCalibur, Becton Dickinson, Franklin Lakes, NJ, USA).

### 4.5. TUNEL Assay

The TUNEL assay was performed to detect apoptosis in CRC cells treated with melittin. Cells were seeded in laser confocal microscope-compatible Petri dishes and treated with melittin for 24 h. After treatment, the cells were fixed with 4% paraformaldehyde for 30 min at room temperature and permeabilized with 0.2% Triton X-100 for 15 min. The TUNEL reaction mixture was prepared according to the manufacturer’s protocol (Elabscience, Wuhan, China) and applied to the cells, which were incubated at 37 °C for one h in the dark. Post-incubation, the cells were washed three times with PBS and counterstained with DAPI for 5 min to visualize nuclei. Apoptotic cells were identified by fluorescence microscopy using a laser confocal microscope (Nikon eclipse, Nikon, Tokyo, Japan), with green fluorescence indicating TUNEL-positive cells and blue fluorescence showing DAPI-stained nuclei. The results were analyzed and documented using Nikon’s NIS-Elements C software (Nikon, Japan).

The TUNEL assay was applied to paraffin-embedded sections to analyze apoptosis in tumor tissues. Tissue sections were treated with proteinase K and permeabilized with Triton X-100, followed by incubation with the TUNEL reaction mixture for 2 h at 37 °C. Apoptotic cells in the tissue samples were visualized using fluorescence microscopy (Nikon Eclipse TI-SR, Nikon, Japan).

### 4.6. ROS Assay

The intracellular ROS levels were measured using the 2′,7′-dichlorodihydrofluorescein diacetate (DCFH-DA) fluorescence probe (ROS Assay Kit; Beyotime Co., Ltd., Nanjing, China). Cells were seeded in 6-well plates and treated with melittin for 24 h. Next, 1 mL of diluted DCFH-DA (1:1000 dilution in serum-free medium) was added to each well, followed by incubation at 37 °C for 30 min. After washing with a serum-free medium, ROS levels were visualized and imaged using a fluorescence microscope (Nikon Eclipse, Nikon, Japan).

### 4.7. Mitochondrial Membrane Potential Assay

Mitochondrial membrane potential was evaluated using the JC-1 Mitochondrial Membrane Potential Assay Kit (Beyotime Co., Ltd., Nanjing, China). Cells were seeded in 6-well plates and treated with varying concentrations of melittin. After 24 h, JC-1 staining solution was added to the cells according to the manufacturer’s instructions, followed by incubation at 37 °C for 30 min. The cells were washed and resuspended in the buffer for flow cytometry analysis of ΔΨm (FACSCalibur, Becton Dickinson, Franklin Lakes, NJ, USA).

### 4.8. Wound-Healing Assay

The migration ability of the CRC cells was assessed using a wound healing assay with 2-well cell culture inserts (ibidi corp., Kirchzarten, Germany). The inserts were placed in 6-well plates, and cells were seeded into the compartments. Once the cells reached 90% confluence, the inserts were removed to create a wound of 500 μm, followed by washing with PBS. Various concentrations of melittin were added, and the cells were incubated for 24 h. Images of the wound area were captured at 0 and 24 h using an optical microscope (Nikon, Japan).

### 4.9. Transwell Migration and Invasion Assay

Cell migration and invasion were evaluated using Transwell inserts with 8 μm pores (Corning Costar Corp., Tewksbury, MA, USA). For the migration assay, 5 × 10^5^ cells were seeded in the upper chamber with serum-free medium, while McCoy’s 5A medium with 10% FBS was placed in the lower chamber. After 24 h of melittin treatment, non-migrated cells on the upper surface were removed, and the migrated cells were fixed with 4% paraformaldehyde and stained with 0.1% crystal violet. Cells were imaged and counted under an optical microscope (Nikon, Japan). For the invasion assay, the upper chamber was coated with a mixture of Matrigel (BD biosciences, USA) and McCoy’s 5A medium (1:8 ratio), and the same procedure was followed.

### 4.10. Western Blotting

After treatment with melittin for 24 h, cells were lysed using RIPA buffer containing protease and phosphatase inhibitors (Roche, Basel, Switzerland). Protein concentrations were determined using a BCA Protein Assay Kit (Beyotime Co., Ltd., Nanjing, China). Equal amounts of protein samples were separated by SDS-PAGE (12% gel), transferred onto PVDF membranes (Millipore, Billerica, MA, USA), and blocked with 5% skim milk. The membranes were incubated overnight with primary antibodies at 4 °C, washed, and then incubated with AP-conjugated anti-rabbit secondary antibody. Protein bands were visualized using a chemiluminescence detection system (Millipore, Billerica, MA, USA), and the bands were analyzed and quantified using ImageJ 6.0 software (Bethesda, Maryland, USA), with the band of the reference protein (*β*-tubulin) serving as the standard. The relative intensity of the target protein was compared to that of the reference protein to calculate the relative expression level of the target protein. The antibodies’ information was described as follows: *β*-tubulin (ab179513, Abcam), Caspase 3 (ab32351, Abcam), Cleaved-Caspase 3 (ab2302, Abcam), Caspase 7 (12827, CST), Caspase 9 (ab202068, Abcam), Bcl-2 (ab182858, Abcam), Bax (ab32503, Abcam), Cytochrome C (ab133504, Abcam), AIF (ab32516, Abcam), Smac/Diablo (ab32023, Abcam), Endo G (ab76122, Abcam), MMP 2 (ab92536, Abcam), MMP 9 (10375-2-AP, Proteintech), E-Cadherin (ab76055, Abcam), N-Cadherin (ab18203, Abcam), Vimentin (5741T, CST), Snail (3879, CST), Twist1 (46702S, CST), Slug (9585T, CST), *β*-catenin (ab223075, Abcam), Axin2 (ab109307, Abcam), Cyclin B1 (ab32053, Abcam) (Abcam, Cambridge, UK) (CST, Danvers, MA, USA) (Proteintech, Wuhan, China).

### 4.11. Animals and Treatments

All animal procedures were approved by the Institutional Animal Care and Use Committee of Zhejiang Chinese Medical University (IACUC-202406-05) and implemented according to the guidelines of the Laboratory Animal Research Center of Zhejiang Chinese Medical University (Certificate No. SYXK, Zhejiang, 2021-0012, China). Male BALB/c nude mice (4–6 weeks old, 18–20 g) were used for in vivo studies. Mice were maintained in a sterile temperature-controlled room under a 12 h light–dark cycle condition, with a standard rodent chow diet and ad libitum access to water. The method of euthanizing mice used was carbon dioxide asphyxiation.

Subcutaneous xenograft tumor models were established by injecting 1 × 10^7^ HCT116 cells into the right flank. Once the tumors reached 80–100 mm^3^, the mice were randomly assigned to three groups: control (PBS), low-dose melittin (1 mg/kg), and high-dose melittin (2 mg/kg). Tumor volume was measured every two days, and after 10 days, mice were sacrificed, and tumors were harvested for further analysis. The tumor volume (V) was calculated using the formula V = {[(W)2 × L]/2}, where ‘W’ represents the width (shortest tumor diameter), and ‘L’ stands for the length (longest tumor diameter).

For the lung metastasis model, 4 × 10^6^ HCT116^luc^ cells were injected intravenously into nude mice. Mice were randomized into control (PBS) and melittin-treated groups (200 μg/kg and 300 μg/kg), with treatment administered every other day following detection of pulmonary metastasis by in vivo bioluminescence imaging (Xenogen IVIS-200, Waltham, MA, USA). After the final imaging, the mice were sacrificed, and lung tissues were collected for analysis.

### 4.12. Histopathology H&E Staining

Tumor tissues were fixed in 4% paraformaldehyde, dehydrated, and embedded in paraffin. For histological analysis, sections (4 μm) were stained with hematoxylin and eosin (H&E). The cell morphology and inflammatory infiltration were examined under an optical microscope (Nikon, Japan).

### 4.13. Immunofluorescent Staining and Immunohistochemistry (IHC)

For immunofluorescence, paraffin sections of tumor tissues were blocked with BSA, followed by incubation with primary antibody at 4 °C overnight. After washing, sections were incubated with CY3-labeled secondary antibodies and stained with DAPI. Images were captured using a fluorescence microscope (NIKON ECLIPSE C1, Nikon, Japan).

For IHC, antigen retrieval and blocking were followed by incubation with primary antibodies. The samples were incubated with HRP-conjugated secondary antibody, and DAB staining was performed. Images were captured using an optical microscope (Nikon, Japan).

### 4.14. In Vitro Angiogenesis Assay

Tube formation by HUVECs was assessed on Matrigel-coated 96-well plates. Matrigel^®^ Matrix (BD 356234, BD biosciences, USA) was prepared by allowing it to thaw overnight at 4 °C. Subsequently, 50 μL of Matrigel was added vertically into each well of a 96-well plate, followed by incubation at 37 °C for 1 h to allow polymerization. HUVECs (2 × 10⁴ cells/well) were seeded and treated with melittin for 16 h. Tube formation was monitored, and images were captured using an inverted microscope (Nikon, Japan). VEGF secretion was quantified using a Human VEGF-A ELISA Kit (Elabscience, Wuhan, China) following melittin treatment.

### 4.15. Statistical Analysis

All statistical analyses were performed using one-way ANOVA, utilizing SPSS 25 software (SPSS Inc., Chicago, IL, USA). Data were presented as means ± SEM. Differences were considered significant at *p* < 0.05. Graphs were created using GraphPad Prism 8 (GraphPad Prism, La Jolla, CA, USA).

## Data Availability

The data presented in this study are available on request from the corresponding author.

## Supplementary Materials

The following supporting information can be downloaded at: https://www.mdpi.com/article/10.3390/ijms252111686/s1.
