# Supplementary material for: Melittin Inhibits Colorectal Cancer Growth and Metastasis by Ac-Tivating the Mitochondrial Apoptotic Pathway and Suppressing Epithelial–Mesenchymal Transition and Angiogenesis
Source: Int J Mol Sci. 2024 Oct 30;25(21):11686. doi: 10.3390/ijms252111686 (PMC11546240; doi:10.3390/ijms252111686)
Supplement: Supplementary file 1 [file ijms-25-11686-s001.zip › ijms-3293099-supplementary.pdf]

Supplementary Information

Figure S1. Melittin induces apoptosis in CRC cells by activating the caspase cascade. (A). The expression levels of Caspase 3, Cleaved-caspase 3, Caspase 7, Cleaved-caspase 7, and Caspase 9 in HCT116 cells; (B). The expression levels of Caspase 3, Cleaved-caspase 3, Caspase 7, Cleaved-caspase 7, and Caspase 9 in HT29 cells. Statistical significance: *, $p < 0.05$, **, $p < 0.01$, ***, $p < 0.001$.

A

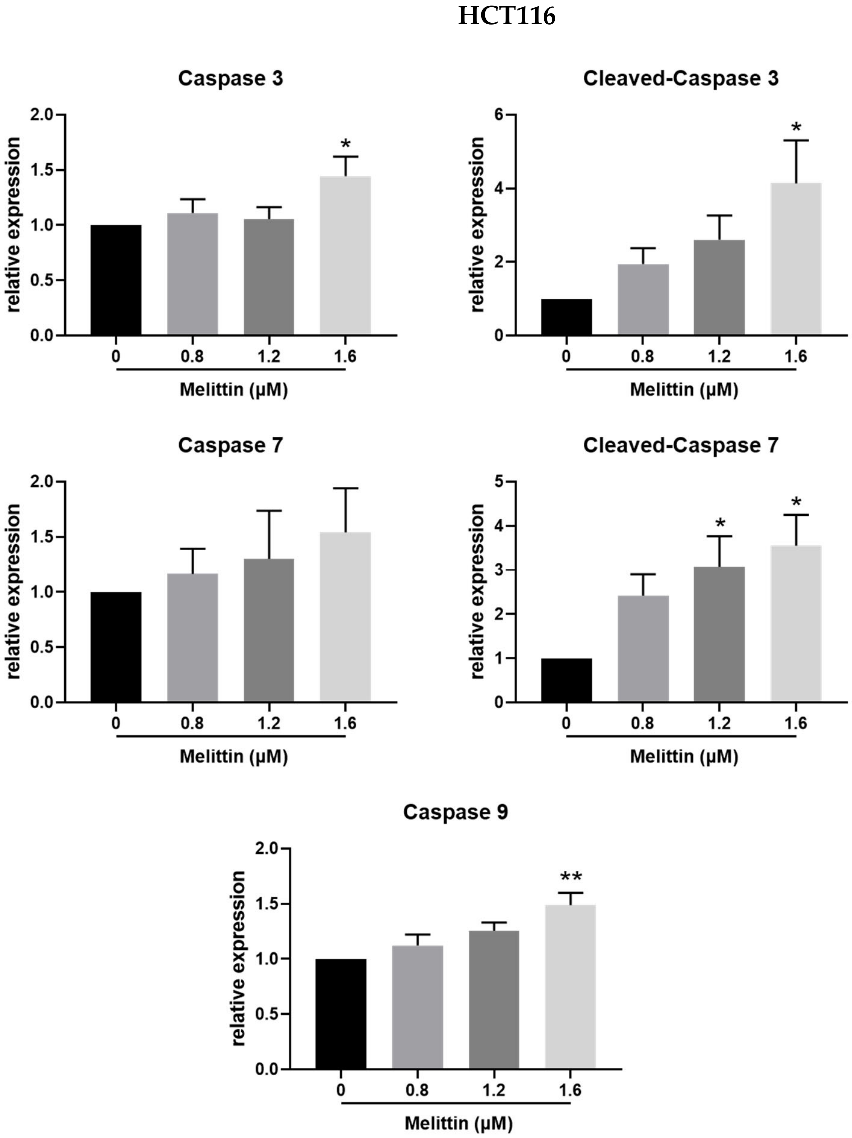

Figure S1. continued

B

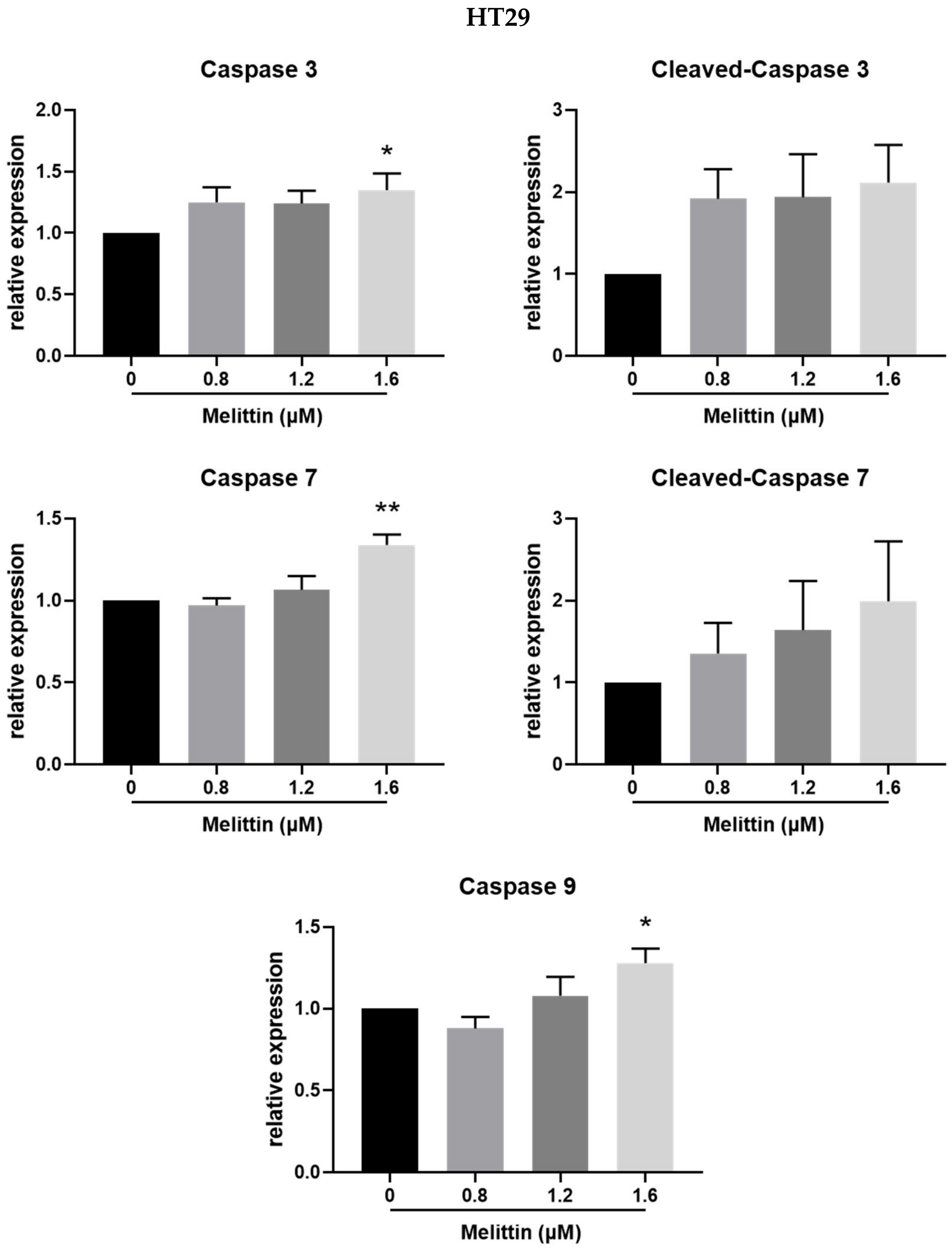

Figure S2. Melittin suppresses tumor growth in a subcutaneous heterograft tumor model using BALB/c nude mice. After treatment with melittin, the tumor volume in mice significantly decreased, with a more pronounced effect in the high-dose (2 mg/kg Melittin) group.

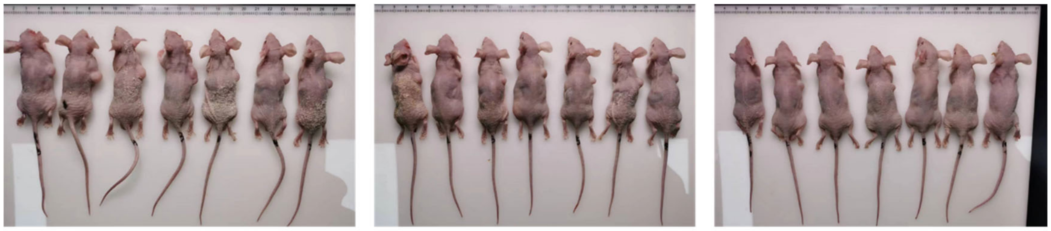

Vehicle | **1 mg/kg Melittin** | **2 mg/kg Melittin**

Figure S3. Melittin induces mitochondrial pathway-mediated apoptosis *in vitro*. (A). The expression levels of Bax, Bcl-2, Cytochrome C, AIF, Endo G, Smac/Diablo in HCT116 cells; (B). The expression levels of Bax, Bcl-2, Cytochrome C, AIF, Endo G, Smac/Diablo in HT29 cells. Statistical significance: *, $p < 0.05$, **, $p < 0.01$, ***, $p < 0.001$.

A

HCT116

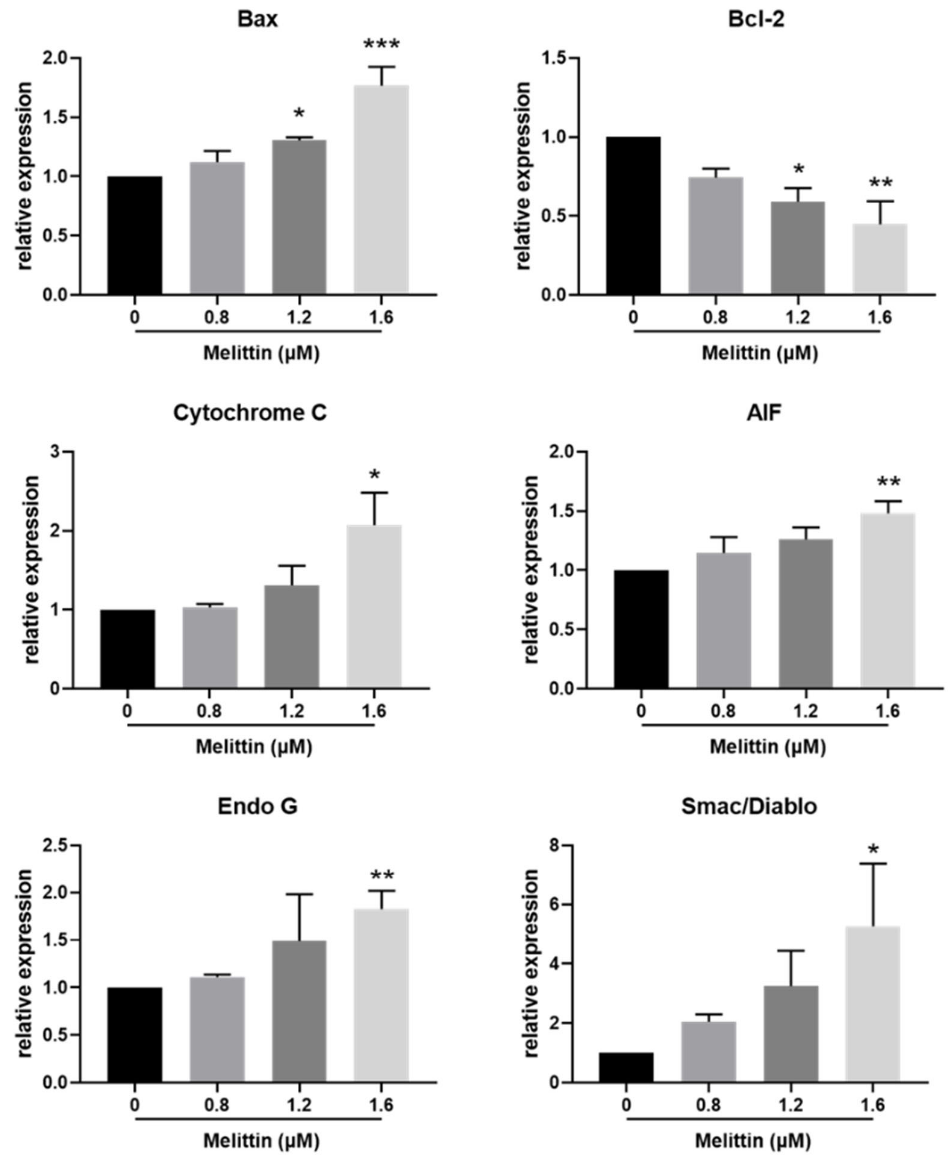

Figure S3. continued

B

HT29

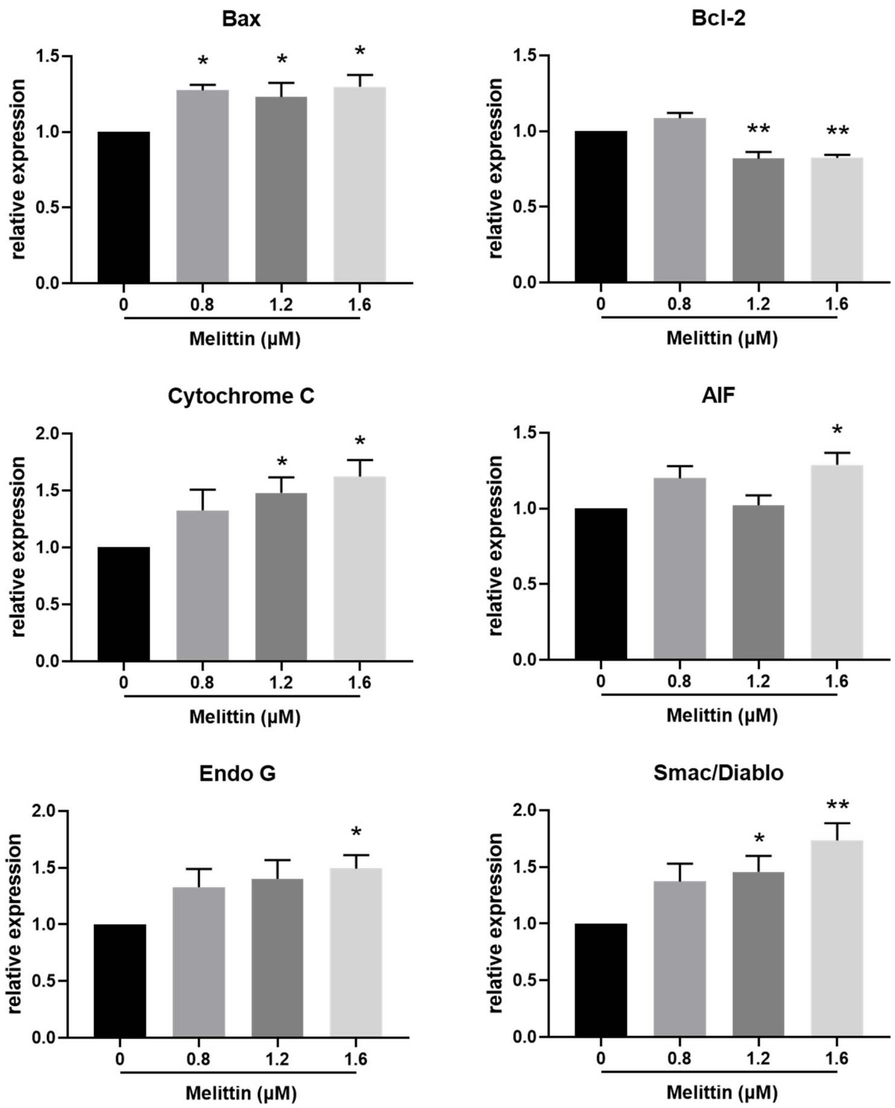

Bax
Bcl-2
Cytochrome C
AIF
Endo G
Smac/Diablo
relative expression
Melittin (μM)
0
0.8
1.2
1.6

Figure S4. Melittin reverses EMT by inhibiting the *β*-catenin signaling pathway *in vitro*. (A). The expression levels of MMPs and EMT-related proteins in HCT116 cells; (B). The expression levels of MMPs and EMT-related proteins in HT29 cells. Statistical significance: *, $p < 0.05$, **, $p < 0.01$, ***, $p < 0.001$.

A

HCT116

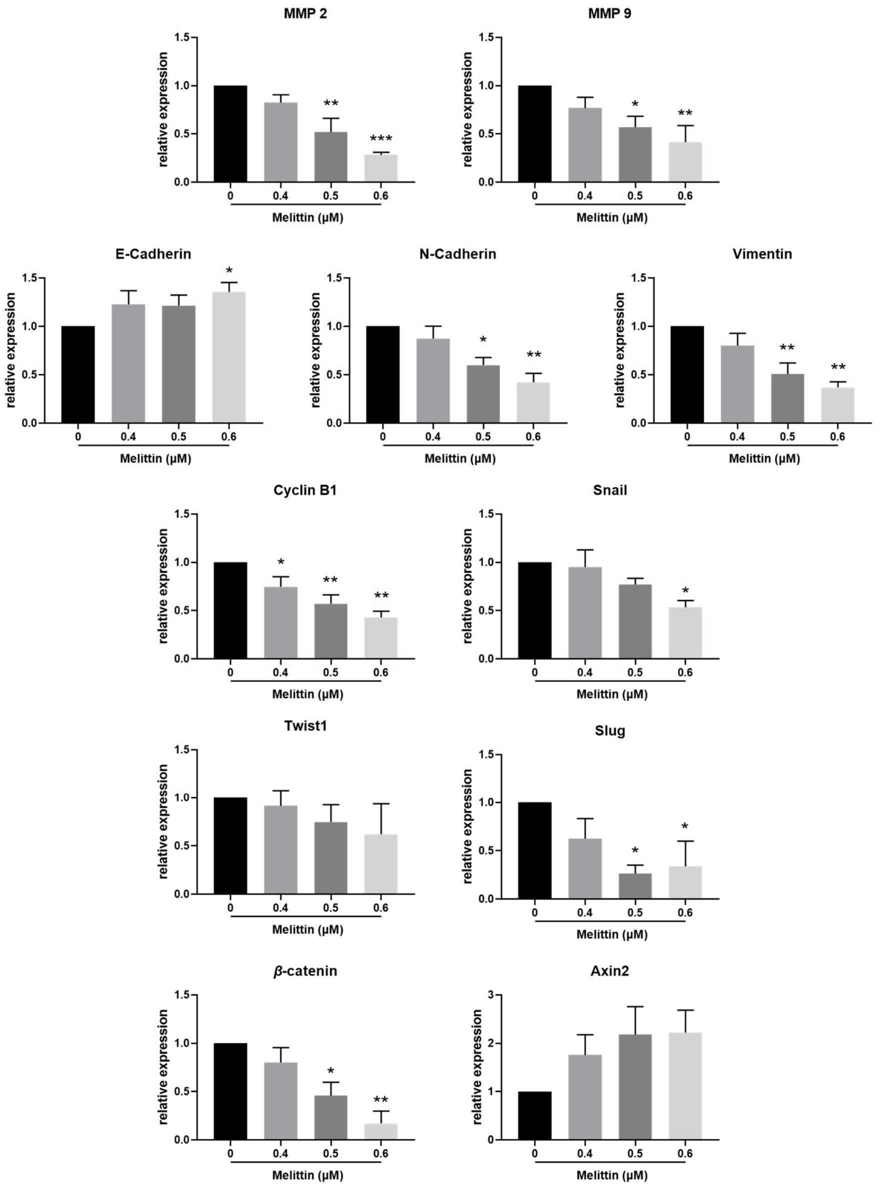

Figure S4. continued

B

HT29

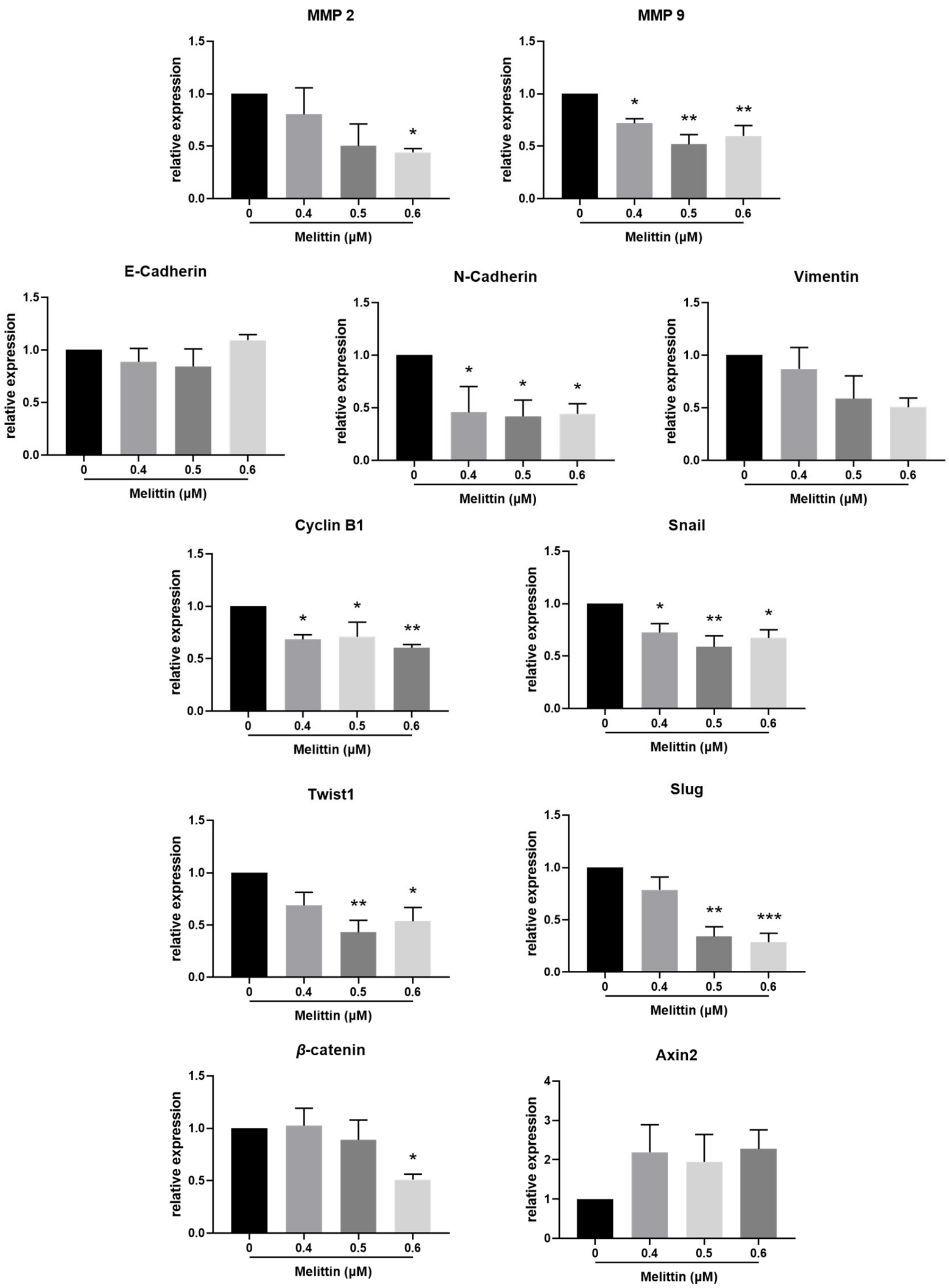
MMP 2
MMP 9
E-Cadherin
N-Cadherin
Vimentin
Cyclin B1
Snail
Twist1
Slug
β-catenin
Axin2
relative expression
Melittin (μM)
0
0.4
0.5
0.6
0.0
0.5
1.0
1.5
*
**
***
